# Intermittent food allergy

**DOI:** 10.1186/2045-7022-1-S1-P68

**Published:** 2011-08-12

**Authors:** Gabriel A Sosa, Maria Luisa Sanz

**Affiliations:** 1 Hospital La Zarzuela, Alergia, Madrid, Spain; 2 Clinica Universitaria De Navarra, Laboratorio de Alergia, Pamplona, Spain

## Introduction

To my opinion, some patients may have reversible tolerance to food allergens. This tolerance that might appear or disappear can be related to the presence of triggers that activate the mast cell, and include exercise, NSAIDs, alcohol or stress.

Different authors have already stated that food allergy can be activated by NSAIDs, exercise, alcohol or stress. These authors demonstrated increase of food absortion as an explanation.

It can also suggest that these triggers lowered threshold for the release of cytoplasmatic mediators from mast cell As we can not work with mast cell we did it with basophils and we evaluate the effect of NSAID on basophile response to food allergens using BAT.

## Methods

We present two patients who presented intermittent clinical symptoms of food allergy, only when the involved food allergen was eaten, simultaneously to NSAID treatment or exercise practice. These two cases tolerate both NSAID, exercise, or the implicated food if they were separate allergens. The Basophile Activation Test (BAT) by flow cytometry, previously reported as an Ag-specific in vitro diagnosis method, was then performed in both circumstances.

## Results

In both patients, BAT showed that CD63 expression by flow cytometry after in vitro stimulation with ASA and culprit food were lower than when both allergens were presented together.

Case 1 food+ASA 42% basophil activation, food alone 28% Case 2 food+ASA 91% basophil activation, food alone 84%.

## Conclusions

Food allergy commonly is not seen as an intermittent phenomenon, but I think some time it is. BAT could be a useful technic for evaluate these, also could be provocation test but it is dangerous.

The paradigm in food allergy is provocation test but we would have to change the paradigm as the patient tolerated food unless we add the trigger.

